# Supplementary material for: Clinical Link between the BARD Score at Diagnosis and Mortality during Follow-Up in Patients with Antineutrophil Cytoplasmic Antibody-Associated Vasculitis
Source: J Clin Med. 2023 Aug 31;12(17):5679. doi: 10.3390/jcm12175679 (PMC10488870; doi:10.3390/jcm12175679)
Supplement: Supplementary file 1 [file jcm-12-05679-s001.zip › jcm-2580475-supplementary.pdf]

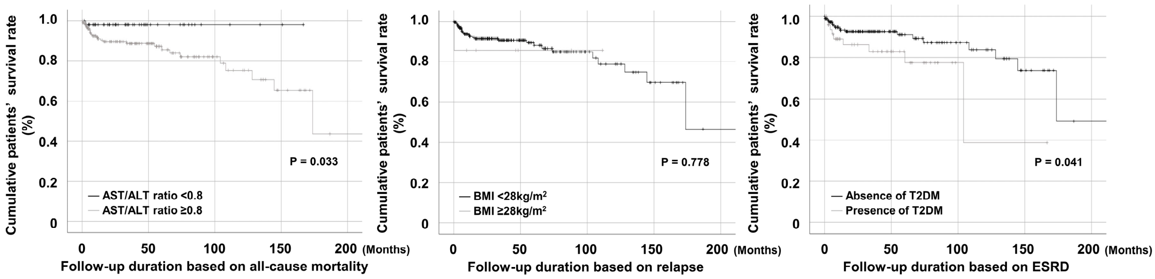

Figure S1: Effect of the parameters of the BARD formula on cumulative patient survival rates in AAV patients.
